# The Effect of Trust on Gaze-Mediated Attentional Orienting

**DOI:** 10.3389/fpsyg.2020.01554

**Published:** 2020-07-15

**Authors:** Mariapaola Barbato, Aisha A. Almulla, Andrea Marotta

**Affiliations:** ^1^Cognition and Neuroscience Research Laboratory, Department of Psychology, Zayed University, Dubai, United Arab Emirates; ^2^Department of Experimental Psychology and Physiology of Behaviour, University of Granada, Granada, Spain

**Keywords:** trustworthiness, trust, gaze-cueing, gaze-cueing effect, attentional orienting

## Abstract

The last two decades have witnessed growing interest in the study of social cognition and its multiple facets, including trust. Interpersonal trust is generally understood as the belief that others are not likely to harm you. When meeting strangers, judgments of trustworthiness are mostly based on fast evaluation of facial appearance, unless information about past behavior is available. In the past decade, studies have tried to understand the complex relationship between trust and gaze-cueing of attention (GCA) (i.e., attentional orienting following another person’s gaze). This review will focus on the studies that used a gaze-cueing paradigm to explore this relationship. While the predictivity of the gaze-cue seems to consistently influence trustworthiness judgments, the impact of trust on gaze-cueing is less clear. Four studies found enhanced gaze-cueing effects with trustworthy faces; one found stronger effects of gaze-cueing with faces associated with undesirable behavior, but only when the observer’s personal evaluations were taken into account. Four studies did not observe an effect of trust on gaze-cueing. Overall, studies have highlighted the complexity of this relationship, suggesting that multiple factors (including age, gender, the characteristics of the observer, and whether or not a threat is perceived) are likely to intervene in the interplay between trust and gaze-triggered attentional orienting. After discussing results in the context of existing theories of gaze-cueing and trust, we conclude that further investigation is needed to better understand this relationship and the contribution of social factors to attentional shifts guided by gaze.

## Introduction

The last two decades have witnessed growing interest in the study of social cognition, the mental operations that underlie social interactions. Social cognition is described as a multidimensional construct including mental-state attribution, emotion recognition, social perception, and social judgments. Several studies have shown that when meeting strangers, we tend to draw social judgments about their traits, qualities, or intentions, often based on facial appearance and after a period as short as 100 milliseconds (Willis and Todorov, 2006). Notably, we are particularly fast (33 ms) at deciding whether an individual looks trustworthy (Todorov et al., 2009). Although there is no universal definition of trust, judging someone as trustworthy is generally understood as believing that this person would not damage or harm you when given a chance (Gambetta, 1988). It therefore does not surprise that trust judgments are performed in such short time: detecting potentially malicious intents in others can have high protective value. In the absence of additional information about other individuals, such as how they behaved in the past, first impressions based on facial appearance represent our best chance to detect others’ intentions. Despite not being predictive of actual behavior, traits like higher inner eyebrows, more pronounced cheekbones, and taller foreheads represent the physiognomic features of what is considered a typical trustworthy face (Oosterhof and Todorov, 2008; Todorov et al., 2008; Ma et al., 2015).

Responding to a stimulus that might represent a threat requires full attention to the stimulus. Recently, studies have started to focus on the relationship between trust and the mechanisms underlying the orienting of spatial attention. Specifically, they have focused on gaze-cueing of attention (GCA) – the orienting of attentional resources in response to another person’s eye-gaze direction. Attentional orienting mediated by another person’s gaze was first described by the early study of Scaife and Bruner in which infants were found to follow the gaze of the experimenter sitting in front of them (Scaife and Bruner, 1975). It is thought to develop early in life (Jessen and Grossmann, 2020) and to play a critical role in the development of more complex aspects of social cognition such as mental-state attribution (Baron-Cohen, 1995). There is general agreement that this mechanism is fundamental for human cooperation and for detection of deceptive behavior. By following the gaze of another person to a (potentially) salient or threatening target, we indirectly acquire information about the other person’s dispositions or intentions toward us.

To learn about the effects of GCA, studies typically use an adaptation of the classic spatial cueing paradigm (Posner, 1980) known as gaze-cueing paradigm (Driver et al., 1999). In this paradigm, participants sit in front of a computer screen where a face is presented, gazing at either the left or the right side of the screen. Participants are instructed to fixate the center of the screen and ignore the eye-gaze when responding to the abrupt onset of a target that could appear on either the same or different side of the screen. Using this paradigm, studies have consistently found that at short cue-target intervals (stimulus-onset asynchrony; SOA), there is an enhanced processing (facilitation effect) for targets presented on the same side of the gaze-cue, and disrupted processing for targets presented on the opposite side, suggesting an automatic shift of attentional resources triggered by the gaze-cue (Frischen et al., 2007).

In recent years, some studies have used a gaze-cueing paradigm to explore the relationship between trust and GCA. Some studies looked at the effect of gaze-contingency on trustworthiness judgments; in other words, they looked at whether trust ratings are modulated by cue predictivity, an effect known as *trust learning*. Other studies focused on the effect of trust on gaze-cueing. Specifically, they used trustworthy and untrustworthy face stimuli as cue, with the goal to understand if GCA is modulated by trust. In the present work, we will review the existing literature exploring the relationship between trust and GCA. To the best of our knowledge, only 13 studies explored, directly or indirectly, the relationship between trust and GCA. Of these, eight studies explored the trust learning effect; seven studies have directly or indirectly tried to understand if trust modulates GCA. While the main goal of the present review is to reach an improved understanding of the influence of trust on GCA, studies exploring the effect of gaze-cueing on trust judgments will be also reviewed, to provide a more comprehensive view of the interplay between these two factors.

## Gaze-Cueing Effects on Trustworthiness Judgments

A first indication of the nature of the relationship between GCA and trust has been provided by a study of Bayliss and Tipper (2006). In a gaze-cuing paradigm, 40 adults were presented with several faces gazing to the left or right. Some faces always gazed toward the target (cooperative gaze), some never gazed to the target (deceptive gaze), and others gazed toward and away from the target in equal proportions (unpredictive gaze). Participants tended to judge faces with cooperative gaze as being more trustworthy than faces with deceptive gaze (trust learning). However, gaze-cueing effects were not influenced by the type of face (cooperative or deceptive). These results were later replicated by the same group with a sample of 72 participants, using emotional stimuli, specifically happy, neutral, and angry faces, as a cue. Results indicated trust learning, particularly for faces displaying positive emotions (Bayliss et al., 2009). With a similar paradigm, Manssuer et al. (2015) have also shown trust learning effects, using electrophysiology observed emotion-related potentials in association with unpredictive gaze-cues, suggesting a role for emotion in trust learning. Moreover, in a later study, Manssuer et al. (2016) further explored the role of emotion using electromyography (EMG) and found evidence indicating that emotional reactions to the predictivity of the cue are necessary in order to observe trust learning. Interestingly, while emotion seems to modulate trust learning, and to influence trust judgments (Oosterhof and Todorov, 2008), its direct effect on gaze-cueing is unclear (Dalmaso et al., 2020). On the other hand, the trust learning effect has been replicated under a variety of conditions (Strachan et al., 2016, 2017, 2020; Strachan and Tipper, 2017). Notably, across studies trust learning was more consistently observed in the form of decreased trust for invalid-cueing faces than as increased trust for valid cues (Strachan and Tipper, 2017), suggesting greater monitoring of deceptive characters over the cooperative ones. It is important to note that the main aim of these studies was to investigate how the predictiveness of eye-gaze direction affects the trustworthiness of faces and that indications about the relationship between trust and gaze cueing effects were only indirectly provided. Nevertheless, these studies have highlighted the strength and automaticity of the gaze-cueing effect, whereby face cues that are 100% deceptive elicit similar attentional orienting as cue that are 100% cooperative, even though they are judged less trustworthy. They also seem to suggest a relative independence of gaze-cueing effects from trust.

## The Effect of Trust on Gaze-Cueing

While trust learning was consistently observed in the literature, the evidence in support of trust modulating GCA is scarce and mixed. To facilitate comparisons, the characteristics of these studies are summarized in Table 1. Previous literature had shown that threatening stimuli tend to capture attention more strongly than non-threatening stimuli (Öhman et al., 2001; Vuilleumier, 2002; Lin et al., 2009), making the idea of greater tendency to follow the gaze of untrustworthy than trustworthy faces plausible. On the other hand, findings highlighting greater gaze-cueing effects for faces with desirable features (e.g., Deaner et al., 2007; Pavan et al., 2011) led some authors to hypothesize that greater attentional orienting should be elicited by trustworthy faces.

**TABLE 1:** The effect of trust on GCA.

**Study**	**Participants**			**Stimuli**				**SOA**	**Interaction**
	***N***	**Age M (SD)**	**Gender (M/F)**	***N***	**Gender (M/F)**	**Database**	**Trust manipulation**	***ms***	***Trust x Cue***
Bayliss and Tipper (2006)	40	20.3 (2.1)	8M/32F	40	M and F*	–	Cue Predictiveness	500	No
King et al. (2011)	24	20.5 (0.88)	24 F	2	Female	–	Vignette	500	No
Petrican et al. (2013)	63	21.3 (3.74)	24M/39F	12	Male	“175 faces manipulated on trustworthiness” (Todorov)	Physiognomy	100/600	No
	69	70.35(5.80)	26M/43F	12	Male	“175 faces manipulated on trustworthiness” (Todorov)	Physiognomy	100/600	Yes, greater gaze-cueing effects with trustworthy faces at long SOA in high cognitive functioning participants
Süßenbach and Schönbrodt (2014)	59	25.4 (8.6)	59F	2	Male	NimStim Face Set (Tottenham et al., 2009)	Vignette	450	Yes, greater gaze-cueing effects with trustworthy faces
Strachan et al. (2017) (Exp. 2)	30	20**	1M/29F	16	Female	KDEF (Lundqvist et al., 1998)	Physiognomy	500	No
Carraro et al. (2017)	55	19.7 (0.86)	15M/40F	6	Male	–	Vignette	200/700	Yes, greater gaze-cueing effects with norm-violating behavior when accounting for personal evaluation.
^†^Jessen and Grossmann (2020)	25	216 (7) days	13M/12F	6	Male	“175 faces manipulated on trustworthiness” (Todorov)	Physiognomy	0	Yes, greater gaze-cueing effects with trustworthy faces
^†^Tummeltshammer et al. (2014) Exp. 1	24	320 (13.40) days	13M/11F	2	Female	–	Cue Predictiveness	500	Yes, longer fixation of cued boxes when the gaze-cue is reliable

SOA, Stimulus Onset Asynchrony; *the ratio between male and female stimuli was not reported in the original manuscript; **standard deviation was not reported in the original manuscript; ^†^these studies measured electrophysiology and eye-movements during overt rather than covert attentional orienting.

After the pioneering study of Bayliss and Tipper (2006), which did not report any effect of trust on gaze-cueing, Kings, Rowe, and Leonards (King et al., 2011) looked at object evaluation as a function of GCA and trust. In this study, 24 female participants were asked to categorize a target item previously cued or ignored by the gaze of one of two possible female sender faces. Before performing the experimental task, the two sender faces were primed as trustworthy or untrustworthy by presenting them to the participants in association to a vignette describing either trustworthy or untrustworthy behavior. Results of this study showed that participants were faster at categorizing targets when these were presented in the location indicated by the cue, regardless of type of cue (trustworthy or untrustworthy).

Later, Petrican et al. (2013) asked a sample of 63 young adult participants from both genders to complete a gaze-cueing task, using male faces that had been manipulated for trustworthiness as gaze-cue stimuli. Their hypothesis was that stronger effects of gaze-cueing should be observed with untrustworthy- than trustworthy-looking faces at short cue-target intervals. Results, however, did not confirm the authors’ hypothesis but rather showed, analogously to what was observed by King et al. (2011), similar attentional orienting in response to the gaze of trustworthy and untrustworthy faces. Notably, an elderly sample of 69 participants (26 males) was also included in this study, with the aim to study age-related bias toward positive (rather than negative) stimuli. In this elderly sample, results highlighted a significant effect of facial trustworthiness on gaze-cueing effects, with greater advantage in response to trustworthy faces compared to untrustworthy.

Another study exploring the relationship between trust and GCA was conducted by Süßenbach and Schönbrodt (2014). Based on evolutionary adaptation theories, the authors hypothesized that humans should have developed the tendency to follow more the gaze of trustworthy than untrustworthy others to minimize chances of deception or danger. Furthermore, they looked at the role of anxiety as a possible moderator of the relationship between trust and GCA. According to previous literature indicating attentional bias toward threatening stimuli in highly anxious individuals (Bar-Haim et al., 2007), the authors hypothesized greater gaze-cueing effects for untrustworthy than trustworthy faces in this population. Participants in this study were asked to complete a standardized scale measuring state and trait anxiety and then to complete a standard gaze-cueing task. After reading a vignette to prime two male face stimuli as trustworthy or untrustworthy, 60 female participants were asked to perform a localization (right/left) of target stimuli that had either a positive or negative valence. Results indicated that faces that had been associated with positive behaviors generally elicited stronger effects of gaze-cueing (i.e., larger facilitation effects). This effect was observed among participants with low (η^2^_G_ = 0.008) and medium level of trait-anxiety (η^2^_G_ = 0.005). Specifically, participants with low level of trait-anxiety demonstrated to follow the gaze of trustworthy but not untrustworthy faces, while those with medium level of trait-anxiety followed the gaze of trustworthy faces more than the gaze of untrustworthy faces. Conversely, individuals with high trait-anxiety equally followed the gaze of trustworthy and untrustworthy faces. The authors concluded that the perceived trustworthiness of an individual influences the extent to which covert attention is oriented in response to this person’s gaze and that this effect might be modulated by the level of trait-anxiety. Nevertheless, the authors acknowledged that their results were only exploratory and cross-validation was needed.

More recently, [Strachan et al. (2017); Experiment 2] selected from a standardized database 16 female faces (8 trustworthy; 8 untrustworthy). Thirty participants (of which 29 females) were asked to perform trustworthiness ratings before and after completing a gaze-cueing task in which the same face stimuli were used as either a valid (i.e., predictive of the target location) or invalid cue. In addition to studying the interaction between trust and GCA, this study also looked at the effect of race (i.e., ingroup/outgroup). Results of this study did not show an effect of race or facial trustworthiness on gaze following, similarly to what had been observed by previous studies (King et al., 2011; Petrican et al., 2013). The authors suggested that the influence of social factors, such as trustworthiness, on gaze-cueing might be contingent on whether or not a threat is perceived. On the other hand, results clearly indicated trust learning, with a smaller effect for outgroup faces.

While these studies all looked at the relationship between trust and GCA directly, a study conducted by Carraro et al. (2017) focused on the effect of a trust-related dimension, personal evaluation of behavior, on GCA. Similar to previous studies, during a learning phase, 6 (male) face stimuli were presented associated with either positive (desirable) or negative (undesirable/norm-violating) behavior. Participants, 73% of which were female, were asked to memorize the association between each face and the corresponding behavior. Then, they performed a gaze-cueing task in which the same face stimuli that had been used in the learning phase were used. After the gaze-cueing task, participants were asked to evaluate the behavior of each character on a 7-point scale from very bad to very good. The authors had hypothesized that participants’ evaluation of the characters’ behavior (i.e., the extent to which participants differentiated between positive and negative behaviors in their evaluations) would play a key role in attentional orienting. Results highlighted a significant interaction between face and gaze-cueing (η^2^*_*p*_* = 0.09) only when participants’ evaluation of the characters’ behavior was taken into account. Specifically, greater gaze-cueing effects were observed when the cue was a norm-violating character. These results seem to suggest that trust-related dimensions such as morality appear to modulate gaze-cueing effects when taking into account certain individual characteristics of the respondents. This result was in disagreement with the results of Süßenbach and Schönbrodt (2014) and with previous literature showing that desirable features of a person increase the tendency to follow his/her gaze direction (e.g., Hungr and Hunt, 2012). The authors, however, suggested that their results support previous evidence indicating that faces can trigger strong gaze-cueing effects when perceived as threatening (Chen and Zhao, 2015).

Finally, two developmental studies including infants provided additional insight on the effect of trust on gaze-cueing. In the first study, Tummeltshammer et al. (2014) recorded ocular movements of 8-month-old infants in response to cooperative or deceptive gaze-cues, preceding the onset of an animated object in four possible boxes located in the four corners of the display. One of the cue stimuli was 100% predictive of the target; the other was predictive only in 25% of the trials. Results showed a significant interaction between face (reliable/unreliable) and cue validity (η^2^*_*p*_* = 0.14), indicating that infants were looking significantly longer at the cued box compared to any other box, but only when the cue was reliable.

In the second study, Jessen and Grossmann recorded, in twenty-five 7-month-old infants, event-related potentials (ERPs) in response to trustworthy/untrustworthy face-cues while using a gaze-cueing task (Jessen and Grossmann, 2020). A total of six emotionally neutral male faces (three trustworthy and three untrustworthy) were used as gaze-cues, followed by images representing toys as targets. Infants were sitting in front of a computer screen while ERPs were recorded. Results of this study have highlighted an interaction between cue validity (i.e., whether or not the cue correctly indicates the target location) and trust (η^2^*_*p*_* = 0.15) at frontocentral electrodes, indicating larger Nc amplitude (i.e., greater allocation of attentional resources) in trustworthy-invalid trials than in untrustworthy-invalid trials, while no influence of trust was observed in the valid trails. The authors interpreted this result as an indication of lack of joint attention in invalid trials with untrustworthy cues. It is important to note that, unlike the above-mentioned literature, these last two studies likely measured overt rather than covert attentional orienting, given infants’ inability to follow the instruction to maintain the gaze on a fixation point at the center of the screen.

Taken together, the mixed results reported in this scarce literature make it difficult to draw any firm conclusions. In the next section, an attempt will be made to identify the methodological differences between studies that possibly contributed to contrasting results.

## Methodological Differences Between Studies and Their Implications

A limited number of studies (8) explored the effect of trust on GCA. Overall, the majority of studies failed to observe a significant interaction between trust and gaze-cueing, and even for the studies whose results highlighted an interaction, findings do not always go in the same direction.

First, to prime cue-stimuli as trustworthy or untrustworthy, two studies manipulated the predictiveness of the gaze cue (Bayliss and Tipper, 2006; Tummeltshammer et al., 2014), some studies relied on physiognomic characteristics of the face stimuli (Petrican et al., 2013; Strachan et al., 2017; Jessen and Grossmann, 2020), and others used vignettes to associate positive or negative *reputation* to the face stimuli (King et al., 2011; Süßenbach and Schönbrodt, 2014; Carraro et al., 2017). All in all, it seems that physiognomy and reputation are equally likely to produce an effect on gaze-cueing. These manipulations, however, are substantially different, one relying on perceptual process and the other on retrieval of previously learned information. Based on evidence showing that learning about someone’s reputation can influence cognitive and affective processing (Qi et al., 2018), one could speculate that attentional orienting in response to somebody else’s gaze should be more influenced by the person’s reputation rather than by facial features. From an evolutionary perspective, Alexander Todorov suggests that, although the characteristics of human faces are optimized for effective communication, for most human history individuals have lived in small-scale societies where information about each other’s past behavior was always available. As a result, relying on physiognomic features for trust judgments has never been necessary while reputation could have played a more important role (Todorov, 2017). Although plausible, this interpretation seems in disagreement with the two studies inducing trust through physiognomy and finding significant effects of trustworthiness on gaze-cueing (Petrican et al., 2013; Jessen and Grossmann, 2020). It is worth noting, however, that these two studies, unlike other studies in this review, did not include young adult samples, but rather included 7-month-old infants (Jessen and Grossmann, 2020) and an elderly sample (Petrican et al., 2013), highlighting the importance of age as another critical factor. Furthermore, it must be noted that Jessen and Grossmann (2020) reported electrophysiological and not behavioral results, which further limits comparisons with previous studies. Nevertheless, some considerations can be made. Being in a preverbal cognitive stage, it is possible that infants seem to strongly rely on facial features when orienting attention in response to other people’s gaze, confirming the importance of gaze as a social cue since early stages of life (Jessen and Grossmann, 2020). One could speculate that reliance on facial features during social interaction decreases as language is acquired, and the tendency to rely on reputation increases, even though results of Tummeltshammer et al. (2014) indicate that infants infer the reliability of another person using information about their past behavior (if available) and not just their facial appearance. Interestingly, Petrican et al. (2013) found greater gaze-cueing effects with trustworthy faces in an elderly sample with higher cognitive functioning and interpreted this result as evidence of an age-specific preference (positivity bias) for positive over negative information in socially relevant situations. These results seem to collide with previous literature reporting age-related decline in gaze-cueing effects (for a review, see Dalmaso et al., 2020) as well as in higher-order dimensions of social cognition, including mental-state attribution and lie detection, especially since these deficits appear to be at least in part independent of changes in cognitive functioning (Sullivan and Ruffman, 2004; Ruffman et al., 2012). One could speculate that, once we can no longer rely on higher-order social cognition, we do not have any other choice than to let perceptual features guide our social behavior. These speculations, however, need to be carefully verified. Notably, studies have also highlighted the importance to take into account the age of the face stimuli when assessing age-related differences in social behavior, including trustworthiness judgments (Johansson-Stenman et al., 2013) and GCA (Slessor et al., 2010; Ciardo et al., 2014). Overall, the role of age of the participants and of the stimuli has been largely neglected in the context of trust and GCA, highlighting the need for further studies.

Regarding the nature of the influence (if any) of trust on GCA, based on the current literature it is not possible to firmly conclude whether it is the gaze of trustworthy or untrustworthy individuals to elicit stronger attentional orienting, even though there seems to be slightly more evidence in support of the former (Petrican et al., 2013; Süßenbach and Schönbrodt, 2014; Tummeltshammer et al., 2014; Jessen and Grossmann, 2020). Importantly, Carraro et al. (2017), although not directly measuring trust, highlighted the role of personal evaluations by reporting increased attentional orienting toward norm-violating characters in individuals who judged the characters’ negative behaviors more negatively. This was interpreted as a possible indication of greater attentional orienting due to increased perceived threat (in agreement with Chen and Zhao, 2015). Süßenbach and Schönbrodt (2014) on the other hand reported that in their study, trait-anxiety modulated the extent to which we tend to ignore the gaze of untrustworthy faces. This seems in agreement with the more careful monitoring of untrustworthy individuals observed in trust learning (Strachan and Tipper, 2017). It is worth noting that, in their analysis, the authors did not test the effect of trait-anxiety as a covariate. Additionally, the three-way interaction between trust, cue validity, and anxiety only approached statistical significance (*p* = 0.06), possibly due to lack of statistical power. Despite these limitations, when dividing their overall sample in three groups based on levels of trait-anxiety, they observed greater effects of gaze-cueing with trustworthy faces in participants with low or medium anxiety. Conversely, individuals with high trait anxiety covertly followed the gaze of both trustworthy and untrustworthy faces indistinctively. The authors attributed this result to a tendency, for anxious individuals, to perceive untrustworthy stimuli as threatening, thus triggering greater attentional orienting (Chen and Zhao, 2015). In this view, these results would not be in complete disagreement with those of Carraro et al. (2017), although these two studies focused on different but related social dimension, which limits the possibility of comparisons. Another important difference between these two studies must be noted: while both studies used vignettes to induce trust/mistrust in the characters, the vignettes in the study of Süßenbach and Schönbrodt (2014) described the untrustworthy character as a violent thief, while Carraro et al. (2017) used less-extreme norm-violating behaviors, which could have resulted in lower perceive threat. All in all, despite that the results of Süßenbach and Schönbrodt (2014) appear in agreement with previous studies reporting a negative association between trait anxiety and trust judgments (Willis et al., 2013), and with evidence of improved working memory processing of untrustworthy faces in highly anxious individuals due to increased sensitivity to potential threats (Meconi et al., 2014), to date this study remains the only one that took into account the moderating role of trait-anxiety in gaze-cueing of trustworthy or untrustworthy faces and one must be cautious when interpreting these results.

Another interesting difference between the studies included in this review involves gender. With few exceptions, studies included prevalently or exclusively female participants. While this is a common limitation in psychology research, what is most interesting is that almost all studies that observed an effect of trust on gaze-cueing used male stimuli as cues (Table 1). According to Süßenbach and Schönbrodt (2014), “*the perceived threat coming from a neutrally looking face arises from a combination of attributed potential danger and perceived untrustworthiness*.” Based on this argument, one could speculate that perhaps studies using female faces as cue could have failed to observe an interaction between trust and gaze-cueing because female faces, even when perceived as untrustworthy, would not be perceived as dangerous by female participants. Conversely, male faces perceived as untrustworthy could also be perceived as dangerous or threatening. This would be in agreement with studies showing that neutral male faces, compared to female, are more likely to be judged as angry and that masculine face features are more likely to be associated with threat (Becker et al., 2007). From an evolutionary perspective, perceiving males as more threatening than females would be advantageous due to obvious gender differences in physical strength (Harris et al., 2016). Masculine faces are also perceived as more dominant (Perrett et al., 1998), and dominance has been associated with stronger gaze-cueing effects (Ohlsen et al., 2013). In this view, studies using male stimuli could have elicited gaze-cueing effects that were generally stronger and more likely to be modulated by trust; however, this would not clarify why the direction of the effect of social evaluations is not consistent across studies. It is also known that men generally tend to trust more than women, but overall women are trusted more than men (Buchan et al., 2008; Manssuer et al., 2016); likewise, gender differences exist in gaze following behavior, where women seem to show greater attentional orienting in response to the gaze of others (Bayliss et al., 2005; Alwall et al., 2010). All in all, it seems crucial that future studies take into account the role of gender in a counterbalanced way and try to clarify the role of perceived threat in gaze-cueing of trustworthy and untrustworthy faces.

Finally, it seems worth noting that studies included in this review were mainly conducted on Western/Caucasian samples, which does not allow to study the influence of cultural differences on both trust and gaze-cueing. Regarding trust, for example, cross-cultural differences have been reported in cooperation, reciprocity, trustworthiness, and trustfulness (Yamagishi and Yamagishi, 1994; Buchan et al., 2002). Similarly, cross-cultural differences in attentional orienting to social stimuli have been reported, indicating that the extent to which individuals rely on contextual information can greatly affect gaze-cueing (Takao et al., 2016, 2018; Dalmaso et al., 2020). Cross-cultural studies could therefore shed more light on the dynamics of the complex relationship between trust and gaze-cueing.

## Final Remarks and Conclusion

Understanding the mental operations that underlie social judgments and their impact on GCA is fundamental to learning more about human social cognition, including how we establish and maintain social relationships, cooperation, and social bonds. There is extensive literature showing that various levels of both gaze-triggered attentional shifts and interpersonal trust can be observed in healthy and clinical populations (e.g., Hooker et al., 2011; Marotta et al., 2017; Sutherland et al., 2020), suggesting that beyond their obvious social nature, these mechanisms are also regulated by biological factors (Shepherd, 2010). An improved understanding of these mechanisms can therefore not only help better understand healthy human interaction but also shed light on the development of clinical symptoms such as social anxiety, social avoidance, hyper sociability, suspiciousness, and paranoia, which can be found in psychiatric and neurological conditions (e.g., Kapur, 2003; Dawson et al., 2004; Shore et al., 2017; Gregory et al., 2019).

Attentional orienting in response to another person’s gaze is social in nature; it is part of daily social interactions, facilitates communication, and can be used with cooperative or deceptive purpose. Therefore, the act of deciding whether someone should be trusted or not should greatly affect gaze-cueing. Remarkably, despite over 20 years of gaze-cueing research, the potential relationship between trust and gaze-cueing has been largely neglected. Nevertheless, other aspects of social interaction have been explored, highlighting the permeability of gaze-cueing effects to multiple social factors (Dalmaso et al., 2020). This has important theoretical implications. Since the pioneering work of Driver et al. (1999), a vast literature has explored the characteristics and time course of the attentional orienting triggered by gaze-cues, and a long-lasting debate has been evolving around the automaticity of this effect. The results reviewed in the present work contribute to this debate. On the one hand, studies demonstrating trust learning in the absence of an effect of trust on gaze-cueing provide support to the automaticity of the gaze-cueing effect (Bayliss and Tipper, 2006; Strachan et al., 2017). On the other hand, the few studies that did find effects of trust on gaze-cueing suggest that albeit automatic, this effect can be modulated by social factors. From an evolutionary standpoint, it seems reasonable to hypothesize that living in groups has, on one side contributed to the development of gaze-cueing responses, and on the other, led to the need for this response to be selective. This idea is supported by the numerous studies showing the mediating effects of socially relevant variables and suggesting that humans do not always follow the gaze of their conspecifics to the same extent (Dalmaso et al., 2020). Importantly, some studies have explored the temporal dynamics of the effects of social variables on gaze-cueing using different SOAs. As pointed out in a recent review, evidence suggests that some of these variables (e.g., mental-state attribution) would rely on top-down control while others would depend on extraction of perceptual features and therefore would modulate gaze-cueing automatically (Dalmaso et al., 2020). As per the temporal dynamics of the modulation of trust on gaze-cueing (assuming that a modulation exists), most studies that reported a modulatory effect of trust used only one SOA, preventing any conclusions on the automaticity of this modulation.

After reviewing the scarce existing literature, a few conclusions can be drawn. First, there is consistent evidence in support of trust learning from gaze-cueing contingency; these findings suggest that attentional shifts evoked by gaze can affect how others are perceived, with cooperative individuals perceived as more trustworthy than deceptive individuals. On the other hand, the effect of trust on GCA is less clear, as the limited studies available have reported either no effect of trust on GCA or effects that seem to go in opposite directions. Notably, the concept of trust is often used with different meanings in different contexts, ranging from being reliable, to cooperative, to non-harmful, and further research needs to clarify which aspect(s) is (are) most relevant in relation to attentional orienting to social stimuli. Additionally, studies have often relied on qualitatively and quantitatively different samples, stimuli, and manipulations and have used a fixed SOA, which does not allow to better understand the temporal dynamics of modulating effects. Regarding samples, studies have included infants, young adults, and older individuals, which makes comparisons difficult due to important cognitive differences between these groups. Nevertheless, investigating the interaction between trust and gaze-cueing across the lifespan would greatly contribute to the understanding of human social interactions. Concerning stimuli, it is known that stimuli of different age and gender are known to trigger different levels of trust and attentional orienting and therefore future studies should carefully control for the role of these factors in a counterbalanced way. Furthermore, the reviewed studies provide preliminary evidence that some individual characteristics of the observer, such as trait-anxiety or personal values, can modulate gaze-triggered attentional shifts. These results, however, await cross validation. In this regard, it appears essential that future studies also clarify whether or not perceiving an imminent threat is a necessary condition to observe an effect of trust on gaze-cueing and whether this effect would show greater sensitivity to the gaze of trustworthy or untrustworthy faces. Notably, the abovementioned factors have emerged from analyzing the limited available literature and are not meant to be exhaustive. On the contrary, there are likely additional factors, biological, social or cultural (e.g., Willinger et al., 2003; Radke et al., 2018; Chang and Baskin-Sommers, 2020), that might affect social evaluations and, in turn, attentional orienting. Further research is therefore strongly encouraged.

## Author Contributions

All authors contributed to the literature review and approved the final version of the manuscript. MB wrote the first and the final version of the manuscript.

## Conflict of Interest

The authors declare that the research was conducted in the absence of any commercial or financial relationships that could be construed as a potential conflict of interest.
